# Estimating poaching risk for the critically endangered wild red wolf (*Canis rufus*)

**DOI:** 10.1371/journal.pone.0244261

**Published:** 2021-05-05

**Authors:** Suzanne W. Agan, Adrian Treves, Lisabeth L. Willey

**Affiliations:** 1 Environmental Studies Department, Antioch University New England, Keene, New Hampshire, United States of America; 2 Carnivore Coexistence Lab, Nelson Institute for Environmental Studies, University of Wisconsin, Madison, Wisconsin, United States of America; Office Français de la Biodiversité, FRANCE

## Abstract

The reintroduced red wolf (*Canis rufus*) population in northeastern North Carolina declined to 7 known wolves by October 2020, the majority of which is due to poaching (illegal killing), the major component of verified anthropogenic mortality in this and many other carnivore populations. Poaching is still not well understood and is often underestimated, partly as a result of cryptic poaching, when poachers conceal evidence. Cryptic poaching inhibits our understanding of the causes and consequences of anthropogenic mortality, which is important to conservation as it can inform us about future population patterns within changing political and human landscapes. We estimate risk for marked adult red wolves of 5 causes of death (COD: legal, nonhuman, unknown, vehicle and poached) and disappearance, describe variation in COD in relation to hunting season, and compare time to disappearance or death. We include unknown fates in our risk estimates. We found that anthropogenic COD accounted for 0.78–0.85 of 508 marked animals, including poaching and cryptic poaching, which we estimated at 0.51–0.64. Risk of poaching and disappearance was significantly higher during hunting season. Mean time from collaring until nonhuman COD averaged 376 days longer than time until poached and 642 days longer than time until disappearance. Our estimates of risk differed from prior published estimates, as expected by accounting for unknown fates explicitly. We quantify the effects on risk for three scenarios for unknown fates, which span conservative to most likely COD. Implementing proven practices that prevent poaching or hasten successful reintroduction may reverse the decline to extinction in the wild of this critically endangered population. Our findings add to a growing literature on endangered species protections and enhancing the science used to measure poaching worldwide.

## Introduction

Red wolves (*Canis rufus)* are endemic to the temperate forests of the eastern United States and are critically endangered, occurring in only one location in the wild where they are the dominant carnivore in the ecosystem [1]. Once extinct in the wild and absent from eastern NC for over 100 years, they were reintroduced from captive born wolves in 1987 beginning with four pairs of wolves into Alligator River National Wildlife Refuge (ARNWR) by the US Fish and Wildlife Service (USFWS), the implementing agency for terrestrial endangered species [2]. Restoring them to their native ecosystems in ecologically functional numbers would meet both the explicit and implicit demands of the Endangered Species Act (ESA) [3] as well as federally established recovery plans [4]. Management includes the 10(j) rule (section 10(j) of the ESA) published in 1995, prohibiting the take of red wolves except for threat to human, livestock, or pet safety along with a 2016 permanent injunction from the US District Court preventing the USFWS from lethally taking red wolves directly or by landowner authorization except as threat to the safety of humans, livestock, or pets [5, 6]. However, the reintroduced red wolf population in NENC has been declining over the last 12 years to a low of only 7 known wolves in 2020 [7].

Even with legal protections, anthropogenic mortality is causing population decline in NENC red wolves [8] and many mammalian carnivores around the world [8]. In studying losses of Mexican wolves *Canis lupus baileyi*, researchers found anthropogenic mortality accounted for 81% of all deaths [9]. With very small populations and limited genetic diversity, the loss of even one red wolf could affect recovery negatively [9]. For the ESA requirement to abate human-caused mortality in endangered species, those human causes should be measured accurately, understood rigorously, and intervened against effectively.

Anthropogenic mortality decreases mean life expectancy for wolves, which has population wide effects. Red wolves rarely live over 10 years in the wild according to the USFWS [10, 11] however, red wolves in NENC live an average of only 3.2 years in the wild with breeding pair duration of only 2 years [10]. This prevents the development of a multigenerational social structure and pack stability, a key factor in preventing hybridization with coyotes [12–16]. Theoretically, wild populations could compensate for anthropogenic mortality through decreases in natural mortality or increases in productivity [17, 18]. However, the strength of compensation in wolf populations is an area of active scientific controversy [19–22]. Also, anthropogenic mortality can be additive or super-additive if a breeder or lactating female is killed, resulting in the death of offspring or through mate turnover in the loss of a breeding male [23]. If there is a large portion of non-breeding adults in the remaining population there is potential for recovery since new breeding pairs could take up residence, however long term compensation for anthropogenic mortality depends on survival of those adults [13, 16, 17, 24]. The Red Wolf Species Survival Plan and USFWS 5-year review both call for removal of threats that have the potential to bring about the extinction of red wolves [25, 26]. Currently, anthropogenic mortality is the leading cause of death for wild red wolves in the endangered NENC population [10]. In the first 25 years of reintroduction, 72% of known mortalities were caused by humans. These included suspected illegal killing, vehicle strikes, and private trapping [2]. Gunshot mortalities alone increased by 275% in the years 2004–2012 compared to 1998–2003 [10, 26] and increased 7.2 times during deer hunting season when compared to the rest of the year. A higher percentage of collared wolves that go missing are unrecovered during this same time as opposed to outside the hunting season [13]. Human-caused mortalities also accounted for 40.6% of all breeding pair disbandment, mostly from gunshots [13], and breeder replacement has decreased since the mid-2000’s [13]. Therefore, precise measurement of mortality using the most current and comprehensive data is critical to understand how sources of mortality influence red wolf population dynamics and its legal recovery under the ESA [10, 27].

The major cause of death in red wolves is poaching [2, 10, 13]. Poaching is a major component of mortality in many carnivore populations [27, 28], but it is still not well understood [29], and disrupts management efforts [30]. As a percent of all mortality, poaching accounted for 24–75% for different carnivore species and areas [31, 32]. More than half of gray wolf mortalities in Scandinavia were the result of poaching, with 66% of those having evidence concealed by the poacher, termed cryptic poaching [33]. In Wisconsin, poaching accounted for 39%– 45% of all gray wolf mortalities over a 32 year period with an estimated 50% cryptic, but this number is believed to be an underestimate because of non-reporting and uncertainty [28–30, 33, 34]. Even a low occurrence of poaching in the red wolf population can have major consequences since unlike gray wolves, red wolves will hybridize with coyotes in the absence of a suitable red wolf mate [13, 16]. The USFWS invested significant resources to stop such hybridization between red wolves and coyotes, however poaching of red wolves continues to increase coyote encroachment [13].

Measuring mortality risk, the proportion of all deaths attributable to a given cause, depends on many factors including the ability to monitor individuals over time. Following individuals with GPS or VHF technologies is the standard method but can be costly, compromise animal welfare, and can create systematic bias when the technology fails, or wolves move out of range [35]. Because killing a red wolf is illegal under the ESA except in case of imminent harm to a human, there may be incentives for poachers to destroy evidence, including radio-collars, which limits the data available to researchers. Destruction of evidence in wolf populations is rarely, if ever, associated with nonhuman COD and never associated with legal human-induced COD [28]. Therefore, measurement error caused by cryptic poaching adds a systematic bias. The possibility that marked animals were poached and monitoring interrupted by destruction of transmitters should be considered in poaching estimates lest we under-estimate the risk of poaching by considering only the subset that is observed by officials because the poacher left a transmitter intact. This underestimation inhibits our understanding of animal life histories, policy interventions, and management actions [29, 36]. Problems such as these can be addressed with models that allow multiple sources of data to inform estimates of variables, including cryptic poaching [28, 33, 37].

Most studies involving mortality data make assumptions that unknown fates resemble known fates [28]. When wolves are legally killed, they are always included in known fates and in calculated mortality risk. Treves et al. [28] tested the hypothesis that in populations where cryptic poaching occurs, known fates do not accurately represent unknown fates causing important losses of information producing systematic error. When they corrected estimates of mortality risk of four endangered wolf populations by excluding legal killing from unknown fates, their estimates of poaching risk were higher than government estimates, which assumed known fates would be representative of unknown fates. For example, when estimates for relative risk from human causes (other than legal) included unknown fates, government reported risk for red wolves was 0.26–0.40 lower than corrected estimates [28]. By accounting for all marked animals, *m* (unknown fates) + *n* (known fates), and estimating cryptic poaching, we extract more information for mortality risk than traditional methods of censoring those marked animals that disappeared [28].

We will adapt the above methods to test the hypothesis that censoring or ignoring marked wolves that disappear under-estimates poaching and loses essential information to produce a systematic bias in conclusions about the NENC red wolves. Here we 1) estimate mortality risk for 5 CODs (legal, poached, vehicle, nonhuman, and unknown) in the red wolf population from 1987–2018, 2) test for association between risk and hunting season, and 3) compare “time to event” for fate unknowns and various CODs. Our analysis adds several years of data not included in prior work and spans a period with a large decline in the wild red wolf population.

## Materials and methods

### Study area

**Fig 1:**
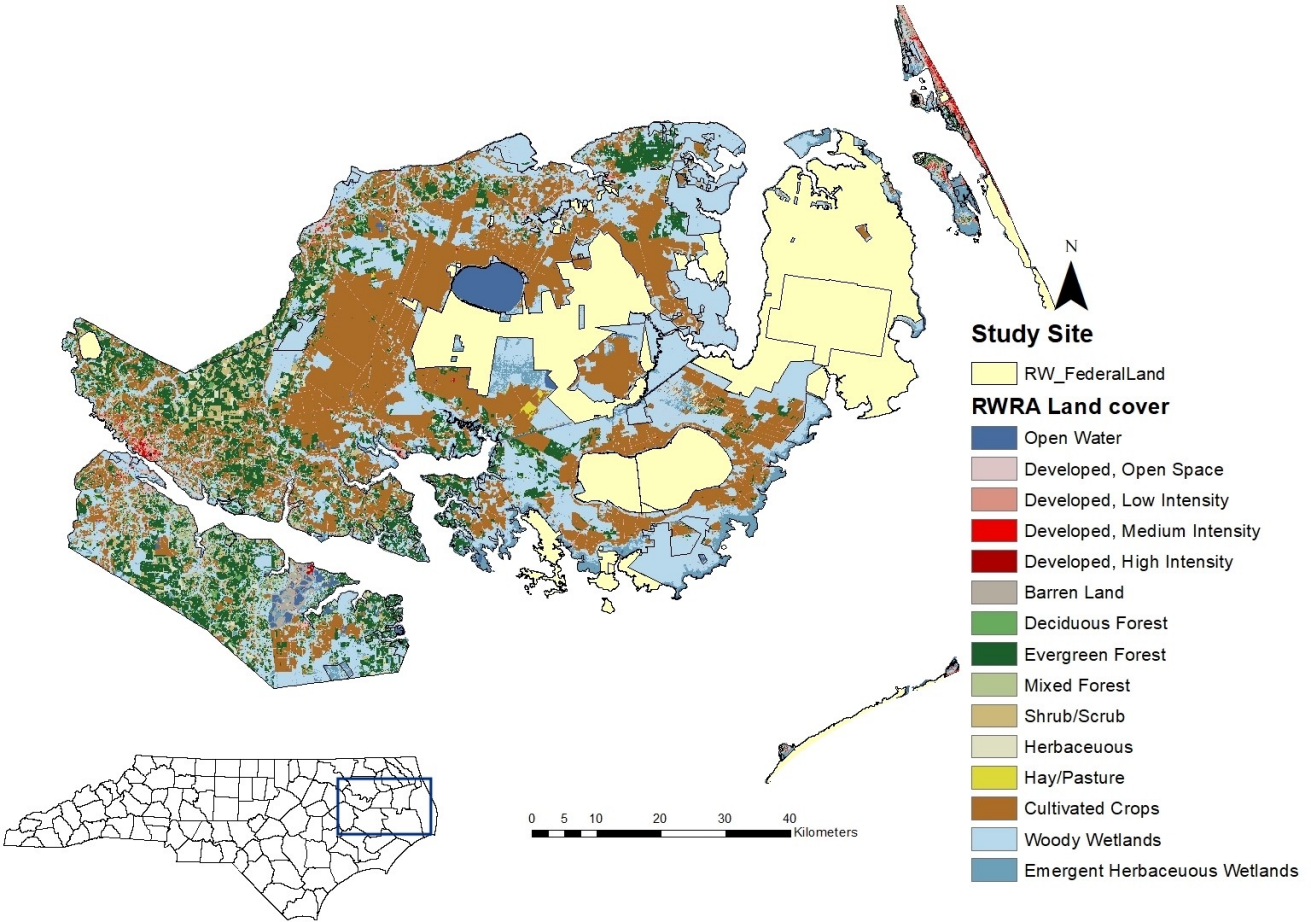
Red wolf recovery area (RWRA) in NENC showing federal and state-owned lands and land cover types [38].

The red wolf recovery area (RWRA) consists of 6,000 km^2^ of federal, state, and private land in five counties on the Albemarle Peninsula, Northeastern North Carolina (NENC): Beaufort, Dare, Hyde, Tyrrell, and Washington. This area includes four USFWS managed National Wildlife Refuges: Alligator River, Mattamuskeet, Pocosin Lakes, and Swanquarter, a Department of Defense bombing range, and state lands (Fig 1). Land cover types for the RWRA include 40% woody wetlands, 26% cultivated crops, 16% evergreen forest, 5% emergent herbaceous wetlands and other minor (less than 5%) land covers of developed, barren land, deciduous forest, mixed forest, shrub/scrub, herbaceous, and hay/pasture [38]. Elevation ranges between 0–50 m and climate is temperate with four distinct seasons [24].

In general, red wolves select for agricultural cover in the recovery area with dispersing individuals using roads and forest-agriculture edges to traverse the landscape [24, 39, 40]. This use of agricultural cover is in contrast to gray wolve use in Wisconsin, which used anthropogenic cover less than expected by chance [34].

### Red wolf sampling

#### USFWS monitoring

Since reintroduction in 1987, the USFWS estimated abundance of red wolves [10] and maintained a database of all known red wolves in the RWRA (Table 1) [41]. Efforts were made to collar every individual, however, radio-collared wolves are not a random sample of all wild red wolves because trapping locations are limited to those areas accessible by USFWS personnel, and pups (<7.5 months) were not radio-collared for their own safety. Unknown wolves may have wandered out of the 5-county recovery area (dispersers), however Bohling (2016) found no red wolves outside the recovery area in their study and so these instances may be rare [42]. We used only adult collared red wolves as detailed life-history data is needed to assign accurate age and risk. Since trapping efforts were conducted for the entire reintroduction period and area, we agree with previous studies that collared red wolves represent the vast majority of this wild population [10]. Red wolves were trapped yearly on federal and state lands as well as private lands with permission from landowners [24]. They were then monitored through weekly radio-telemetry flights [10]. Following Hinton et al. (2016) we define a red wolf year as October 1 –September 30.

**Table 1:** Annual red wolf population size, COD and FU for collared, adult red wolves.

Mortality and FU totals for wolf year Oct 1 –Sept 30							
	Population Est.	Poached	FU	Legal	Collision	Nonhuman	Unknown COD
1987–88	16	0	0	0	2	2	0
1988–89	15	0	0	0	1	2	0
1989–90	31	0	2	0	2	0	0
1990–91	34	0	0	0	2	4	0
1991–92	44	0	0	0	0	0	1
1992–93	67	0	1	0	0	2	0
1993–94	52	0	1	1	4	4	5
1994–95	44	7	2	1	1	2	3
1995–96	52	3	5	1	1	1	3
1996–97	46	0	3	2	3	2	1
1997–98	69	1	7	0	2	1	5
1998–99	90	3	10	2	0	4	2
1999–00	104	6	4	9	1	0	3
2000–01	96–108	4	3	0	2	1	7
2001–02	97–121	10	7	4	4	4	0
2002–03	102–128	5	1	3	3	7	0
2003–04	113–149	4	5	1	5	5	1
2004–05	125–151	7	7	2	2	4	2
2005–06	126–143	7	5	0	4	3	3
2006–07	116–134	9	7	1	3	1	2
2007–08	115–137	9	7	0	3	3	5
2008–09	111–138	5	8	0	3	1	10
2009–10	111–135	8	5	0	3	4	4
2010–11	112–123	6	4	0	3	5	6
2011–12	104–127	10	6	1	1	0	1
2012–13	103–112	12	4	0	3	1	3
2013–14	113–149	11	3	0	2	3	1
2014–15	74	11	4	2	0	0	3
2015–16	45–60	3	0	0	2	0	7
2016–17	20, 25–35	6	3	0	0	1	1
2017–18	19, 23–30	2	1	0	3	0	2

^1^Sources: USFWS database, Hinton et al (2016), and the 2016 Red Wolf Population Viability Analysis [10, 41, 43].

The USFWS database contains information on trapping, tagging, and demographic and spatial information on 810 red wolves including an initial, suspected cause of death (COD), a final, official COD, and necropsy results if performed for each carcass found. These data were collected by the USFWS through field work, reports from trappers, private citizens, and mortality signals from radio-collars [10, 41].

We used population estimates from each year from three sources for red wolf population data: the USFWS database, Hinton et al. (2016), and the 2016 Red Wolf Population Viability Analysis [10, 41, 43]. Population estimates from these three sources were identical until 2000 when they began to differ. We do not know why the variation arose, so when the three sources differed, we presented a range of values from the three sources.

#### Classifying fates

We analyzed data for 508 radio-collared adult red wolves between the years 1987–2018 (63% of all red wolves found in the database; the remaining 37% were pups or uncollared red wolves, which we excluded). The 508 include 312 wolves of known fate and 196 wolves of unknown fate defined later (Table 1).

We reclassified USFWS cause of death for the "known fates" subset into 4 mutually exclusive classes: legal, poached, vehicle and nonhuman (Table 2). Legal refers to legal removal by USFWS or by a permitted private individual; legal is the only perfectly documented COD in our dataset. All other CODs have different amounts of bias depending on cause, which we refer to as inaccurately documented causes of death, following Treves et al. [34]. Because the ESA made it illegal to kill a listed species except in defense of life [44], we define any non-permitted killing of a wolf such as shooting, poison, trapping, etc. as a type of poaching, even if the intended target animal was not a red wolf [34]. We classified as “poached” both the 137 carcasses classified as poisoned, shot, or trapped in the USFWS database and 12 cases the USFWS classified as “suspected foul play”, which included finding a cut collar but no wolf, following Hinton et al. [10] and Treves et al. [34]. We classified “vehicle” separately from “poached” because the driver likely did not plan to kill any animal, following [28]. Finally, “nonhuman” COD include mortality related to intraspecific aggression and health related issues such as disease and age.

**Table 2:** Red wolf fates by our classifications, 1987–2018 for 508 radio-collared adults. The variable *n* (known fate subset) estimates the sum of known COD, and *m* (unknown subset) estimates the sum of FU and unknown COD. FU are those wolves who were radio-collared but were lost to USFWS monitoring. Legal COD was perfectly reported, all other CODs were not.

USFWS official COD or fate	COD for this analysis	Number of marked adults
Management related	Legal	30
Permitted activity		
Gunshot	Poached	149
Poison		
Trapping		
Vehicle	Vehicle	66
Intraspecific	Nonhuman	67
Health/disease		
		**Subtotal for n = 312**
Unknown cause	apportioned between vehicle, nonhuman, poached and cryptic poached	81
Fate unknown (FU)		115
		**Subtotal for m = 196**

Our “unknown fate” subset includes red wolves classified by the USFWS as either unknown COD or fate unknown (FU). FU wolves were lost to USFWS monitoring. Eventually, the USFWS stopped monitoring these collars because they could not locate them through aerial or ground telemetry. This could happen if the collar stopped transmitting and the wolf died undetected by any other means, or if the wolf was killed and the collar was destroyed, referred to as “cryptic poaching” [28, 33]. The USFWS assigned their FU date as the date of last telemetry contact. We included collared red wolves that might still be alive but unmonitored (FU) at the time of this analysis because there are only 7–8 collared wolves known to be alive as of writing and failure to include the rest of the FU that are most likely dead would again overrepresent perfectly reported legal killing [28]. Unknown COD were carcasses found but for which cause could not be determined; some poisons leave no trace or carcasses may decompose in NC heat such that human action is undetectable. Because vehicle collisions may not leave signs of major trauma detectable a week or more after death if decomposition or scavenging destroys the carcass, we consider unknown COD might be assigned to “vehicle”, “nonhuman”, “poached”, or “cryptic poached”. Unknown COD are important to include in our analyses, as discarding them would also overrepresent perfectly reported legal killing [28].

We describe below three scenarios for estimating the number of wolves that died from cryptic poaching among the unknown fates (the sum of unknown COD and FU cases). In short, we expand the notion of cryptic poaching to include marked animals for which illegal killing was possible but could not be confirmed.

The “unknown fate” subset (*m*), including unknown COD and FU (Table 2), contain an unknown number each of nonhuman, vehicle, poached, and cryptic poached CODs. Transmitter failure that leads to FU for a collared wolf evading monitoring while the wolf is alive can happen for any of nonhuman, vehicle and poached CODs and account for a portion of *m*. When a transmitter fails and later the animal was killed illegally, the poacher may not have tried to conceal the evidence. Therefore, some FU should be classified as “poached” rather than “cryptic poached”. In short, not all poaching in FU was cryptic, if the poacher did not intend to conceal evidence. However, when the transmitter is destroyed by the poacher (rather than transmitter failure) or an indetectable method for killing a wolf was used (e.g., poison), then we define it as cryptic poaching. Cryptic poaching never occurs in the known fate subset. Cryptic poaching involves the destruction of evidence [33] distinguishing it from other types of illegal killing that is present in both *m* and *n* (Table 2). By including unknown COD and FU in our “unknown fates” and apportioning some of those “unknown fates” to “poached” and “cryptic poached”, we acknowledge a situation in the NENC that may not be found widely. Namely that some poachers may have thought they were poisoning, shooting or trapping coyotes (including periods of night-time shooting), and left the carcass unwittingly failing to report their error. Therefore, our approach to “cryptic poached” is conservative, allowing for honest error within the illegal CODs. Hereafter we distinguish these two types of poaching in our methods and analysis.

### Estimating risk from different causes of death

Risk is defined as the proportion of all deaths attributable to a given cause. For example, if five out of ten wolf deaths are the result of poaching, then wolves in that population had a poaching risk of 50%. This is different from mortality rate, which is the rate of individuals dying per unit time, therefore the denominator is all wolves living or dead [34]. To estimate risk accurately, we need the denominator to be all collared red wolves (*n + m*, Table 2). With *n* = 312 wolves with information on COD and *m* = 196 wolves without information on COD (Table 2), the denominator for all risk estimates would be 508. See [28] for a full mathematical description of the method. Below we explain how we adapted it for the red wolf dataset.

**Fig 2:**
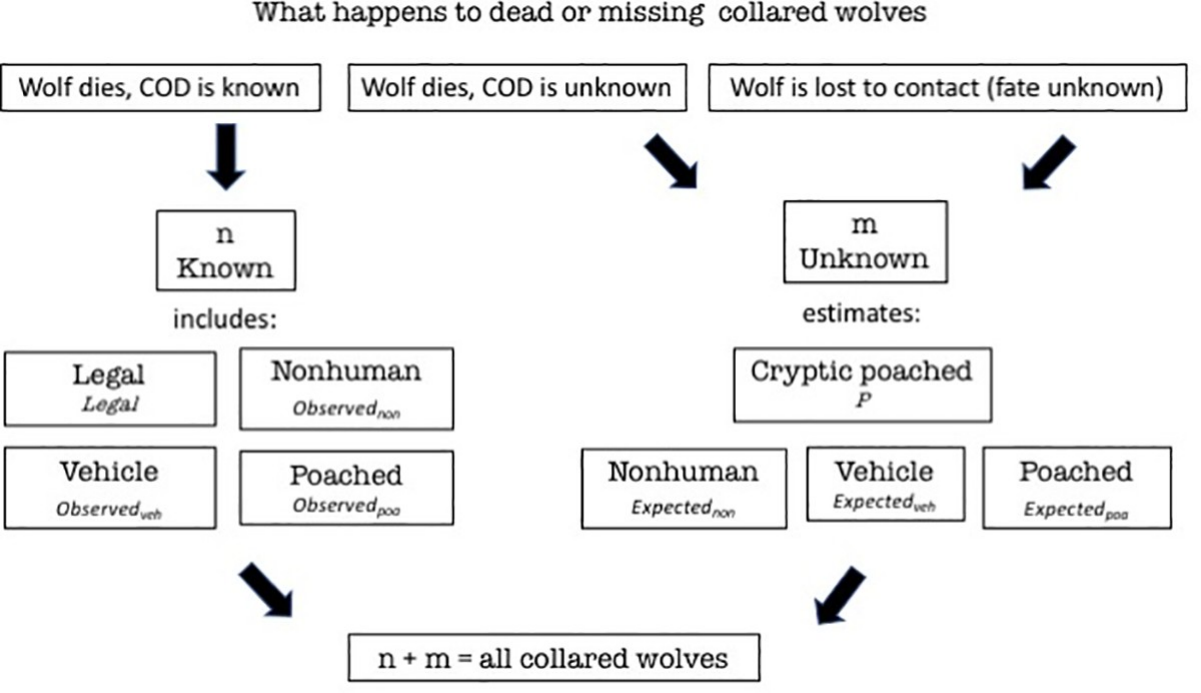
Unknown fates can be estimated. Observed_non_ is the number of marked animals of known fate that died from nonhuman causes. Observed_veh_ is the number of marked animals of known fate that died of vehicle collisions, and Observed_poa_ is the number of marked animals of known fate that died of poaching. Expected_non_ is the number of marked animals of unknown fate expected dead from nonhuman causes, Expected_veh_ is the number of marked animals of unknown fate expected dead from vehicle collision and Expected_poa_ is the number of marked animals of unknown fate expected dead from poaching. P is the estimate of marked wolves dead from cryptic poaching. To account for the possibility of cryptic poaching with transmitter destruction before the wolf meets any other fate, we allocate *m* to the three imperfectly reported CODs. That is why the box for cryptic poaching appears above the others on the right side of the figure.

First, we calculated the risk of legal killing, in a straightforward manner because they are all known fates perfectly reported by definition and there are none in *m*, the unknown fates portion (Fig 2). Therefore, we had to recalculate the risk posed by the 30 cases of legal killing (Table 1) with the denominator, *n + m*, to estimate the risk of legal killing for all collared red wolves at 5.9% (30/508). Without this correction, risk of legal killing in Table 1 would be overestimated at 9.6%. When one over-estimates the risk of legal killing, one underestimates all other imperfectly reported CODs because the total must sum to 100%.

With the straightforward case of legal killing recalculated as explained above, one can more efficiently explain how to account for the imperfectly reported CODs within *m* (Fig 2). The subset of collared wolves in *m* contain only imperfectly reported CODs but an unknown number of each class of nonhuman, vehicle, poached, and cryptic poached.

For our “unknown fate” subset, we turn to estimation techniques whose uncertainty produces bounds on our values. Following the method in [26], we estimate risk more accurately by taking into account how many of *m* to reallocate to our four classes of imperfectly reported CODs. The reallocation step is an estimation procedure with several possible scenarios that apportion different amounts of *m*, depending on assumptions that we explain next. We assumed that if a transmitter was destroyed, that would happen before it failed since failure is rare and would have been unexpected in these wolves, and so when we estimated “cryptic poached”, we deducted the estimate of cryptic poaching from *m* first, before we assign the remainder of *m* to “nonhuman”, “vehicle”, and “poached”.

Previous work [26, 37] assumed the lower bound for estimating cryptic poaching was zero. However, we assumed non-zero cryptic poaching in the NENC red wolf population because we had prior information. At least 23 reported cases in *n* show evidence of attempted and failed concealment of evidence (tampering or damage to the transmitter that did not cause its failure), so the USFWS recovered the collar or carcass even though the poacher tried to conceal it.

The cases of failed concealment included damaged collars found, the dead wolf was found with a damaged collar, or the dead wolf was discovered in a suspicious location with a damaged collar (e.g., dumped in a canal, near a beagle that had also been shot, and one was in the same location as another shot wolf). Salt water can destroy radio-transmitters [45]. Damaged collars included obvious human tampering such as bullet holes and knife cuts. These 23 cases include 12 instances categorized by the USFWS as “suspected foul play” and 11 with suspicious circumstances recorded in field notes [39]. In sum, we inferred that a scenario with zero cryptic poaching was so unlikely as to be discarded and the lower bound of our scenarios should reflect the data more realistically.

Our scenarios triangulate on the probable real value and address uncertainty around a “most likely” outcome as in prior work [43]. Following [26], we used 3 scenarios to estimate “cryptic poached” (P). Scenario 1 assumes that poachers who tamper with evidence are equally successful as unsuccessful, so although the observed risk of cryptic poaching in *n* is zero, the risk of cryptic poaching in *m* should account for successful concealment which we simulate to happen at the same rate as the 23 unsuccessful attempts found in *n*. Therefore, we estimated cryptic poaching as 23*/n* (23/312 = 0.074) meaning P = 0.074 * *m* = 14.4 wolves were successfully concealed and assigned to “cryptic poached”. We treat this as a lower bound because prior studies of cryptic poaching in wolves have estimated the frequency as 50–69% [27, 37] which makes 7.4% seem low.

By contrast our scenario 2 assumed a higher success rate at concealing evidence, in that all FU resulted from cryptic poaching but none of the unknown COD did. While this might over-estimate a few cases of transmitter failure, it might under-estimate cryptic poaching in the unknown COD cases that might include poaching that was concealed by decomposition or untraceable toxins. Therefore, for scenario 2, P = 115 wolves.

For Scenario 3 we rely on the published estimate of cryptic poaching for Wisconsin wolves, of 46%– 54%. We chose the Wisconsin estimate of two available because the other for Scandinavian wolves would imply P > m, a logical impossibility. The Wisconsin estimate yielded P = 149 wolves.

For all scenarios, we subtracted P from *m* first and then estimated the remainder in *m* as the expected numbers for nonhuman, vehicle, and poached as follows:

$$Expected_{non}=\left(m‐P\right)*Observed_{non}/\left(Observed_{non}+Observed_{veh}+Observed_{poa}\right)$$ (Eq 1A)

$$Expected_{veh}=\left(m‐P\right)*Observed_{veh}/\left(Observed_{non}+Observed_{veh}+Observed_{poa}\right)$$ (Eq 1B)

$$Expected_{poa}=\left(m‐P\right)*Observed_{poa}/\left(Observed_{non}+Observed_{veh}+Observed_{poa}\right)$$ (Eq 1C)

Finally we estimate risk for each COD using the expected numbers from 1a-c above and the equations in Table 3. From the two subcategories of poaching, we can estimate total poached (*poached + cryptic poached*).

**Table 3:** Equations used to estimate risk from nonhuman, vehicle and poached CODs.

	Known fates (n)	Unknown fates (m)	Known + Unknown fates (n+m)
Nonhuman	*Observed*_*non*_ /n	*Expected*_*non*_/m	(*Observed*_*non*_ +*Expected*_*non*_)/(n+m)
Vehicle	*Observed*_*veh*_/n	*Expected*_*veh*_/m	(*Observed*_*veh*_ +*Expected*_*veh*_)/(n+m)
Total Poached	*Observed*_*poa*_/n	*Expected*_*poa*_/m	(*Observed*_*poa*_ +*Expected*_*poa*_)/(n+m)

### Timing of death and disappearance

#### Season

We used a binomial test to evaluate whether CODs during the hunting season (141 days from September 12 –January 31, to include all fall and winter hunting of black bear, deer, and waterfowl) was significantly different from the non-hunting season. Since NC issues nonresident hunting licenses, and several large hunting clubs operate within the five-county RWRA, there were annual influxes of hunters. Regulations for hunting coyotes in the RWRA have varied between 2012 and 2015 and since then coyote hunting has been limited to daytime, year-round, with a permit. Using data from 1987–2013, Hinton et al. showed a dramatic decrease in red wolf survival during the months of October through December [10].

#### Individual survival

We estimated the amount of time individual red wolves spent in the wild from their collaring date to the date of death (with COD) or disappearance (FU) at last contact, all in mean days. We expect a systematic under-estimate of time to FU because monitoring periods were presumably independent of the time that a transmitter stopped, so the last contact always preceded COD. This bias would tend to under-estimate the survival time for the imperfectly reported CODs, especially cryptic poaching relative to all other CODs and other imperfectly reported CODs relative to legal. Addressing this bias and conducting a formal time-to-event analysis was beyond our scope.

We conducted all analysis in STATA IC 15.1 for Mac [46].

## Results

### Estimating risk by cause of death (COD)

From 508 adult radio-collared red wolves, we estimated the risk of legal killing as 0.059. Relative risk from human COD other than legal ranged from 0.724–0.787 depending on the three scenarios for level of cryptic poaching (Table 4).

**Table 4:** Risk of legal, nonhuman and other human causes of death for known fates (*n*) and the unknown subset (*m*) using 3 cryptic poaching scenarios (P from Eqs 1A–1C). Total poached is the sum of poached and cryptic poached. Other human is the sum of Vehicle and Total Poached (Table 4 in S1 File).

		Scenario 1, P = 14.4		Scenario 2, P = 115		Scenario 3, P = 149	
	Risk in *n*	*m*	*n+m*	*m*	*n+m*	*m*	*n+m*
Legal killing	0.096	0.000	0.059	0.000	0.059	0.000	0.059
Nonhuman causes	0.215	0.220	0.217	0.098	0.170	0.057	0.154
Other human	0.689	0.780	0.724	0.902	0.771	0.943	0.787
Vehicle	0.212	0.217	0.214	0.097	0.167	0.056	0.152
Total poached	0.478	0.563	0.511	0.805	0.604	0.887	0.635
Cryptic poached	0.000	0.074	0.028	0.587	0.226	0.760	0.293
Poached	0.478	0.489	0.482	0.218	0.378	0.127	0.342

**Fig 3:**
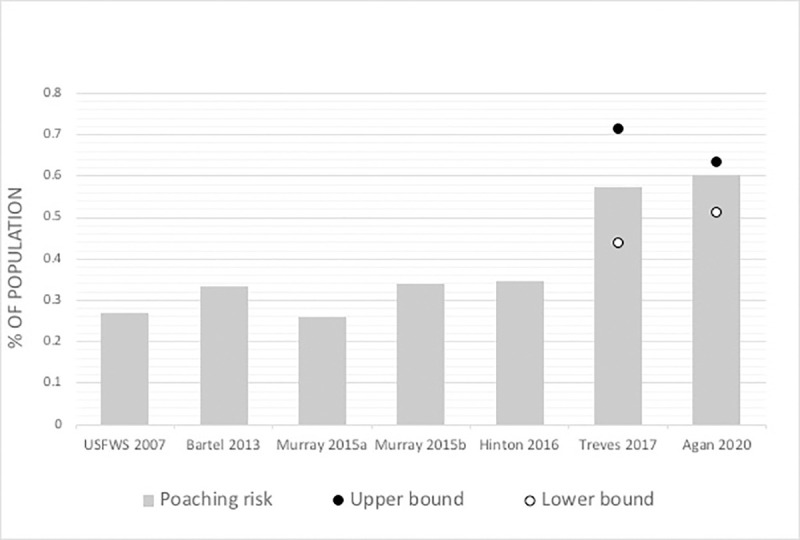
Risk of poaching estimated with different methods from overlapping but different samples of years of the NENC red wolf population. Treves [28] and this study presented 3 scenarios of cryptic poaching with a lower (open circle), middle (bar) and upper (closed circle) bound. Years of data vary by study [2, 10, 26, 47].

In scenario 1 with P = 14.4, we estimated total poaching risk at 0.511 with cryptic poaching accounting for 0.055 of total poaching (Fig 3). In scenario 2 with P = 115, our estimate of total poaching was 0.604. In this scenario, cryptic poaching accounted for 0.375 of total poaching, similar to the Scandinavian estimate [33]. In scenario 3 with P = 149, our estimate of total poaching was 0.635 with cryptic poaching accounting for 0.462 of total poaching as planned by using the Wisconsin estimate.

### Timing of death and disappearance

#### Season

**Fig 4:**
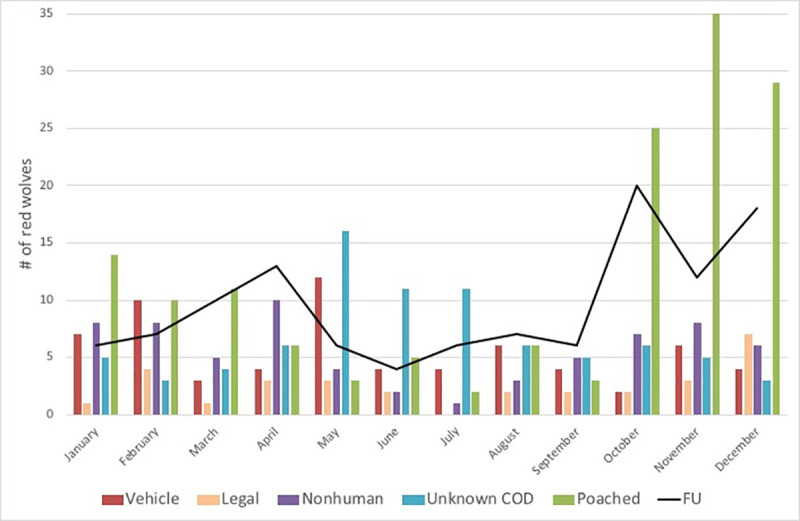
Red wolf mortality each month (bars) compared to fate unknown (FU, black line). FU are represented with a black line because there is more uncertainty with date of disappearance than with date of known deaths. Causes of death include legal, vehicle, poached, nonhuman, and unknown cause of death (COD) (Fig 4 in S1 File).

In our sample of 508 collared, adult red wolves, the three months spanning October to December accounted for 61% of all poached (October *n* = 25, 17%, November *n* = 35, 24%, December *n* = 29, 20%), which a binomial test indicated is significantly higher than expected by chance (25%) (p < 0.001) (Fig 4). The same three months also accounted for 43% of all FU classifications (October *n* = 20, 17%, November *n* = 12, 10%, December *n* = 18, 16%) which is also significantly higher than expected by chance (25%) (p = 0.001). No other CODs were significantly higher during this season.

**Fig 5:**
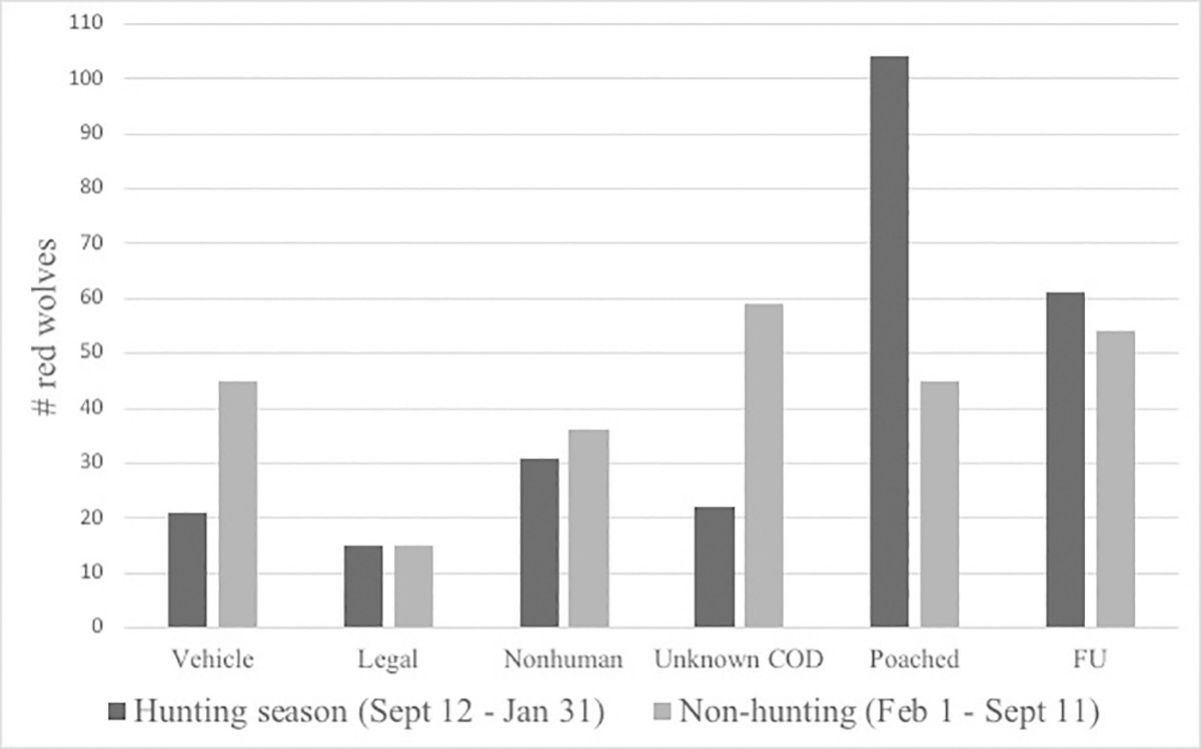
**Red wolf deaths and disappearances (FU) by cause during hunting season (dark gray) and non-hunting season (light gray).** Hunting season is inclusive of all fall/winter hunting including white-tailed deer, black bear, and waterfowl (Fig 5 in S1 File).

We extended our analysis to the full annual hunting seasons of September 12 –January 31 or 141 days. FU and poached occurred significantly more often than expected during hunting seasons, (binomial test for FU: expected 38.6%, observed 61 of 115 or 53%, p = 0.002 and poached was 104 of 149 or 70%, p < 0.001). Unknown COD was significantly lower than expected during hunting seasons (22 of 81 or 27%, p = 0.04) (Fig 5). There were no significant differences in vehicle, nonhuman, or legal COD between the hunting and non-hunting seasons.

#### Time in the wild

**Fig 6:**
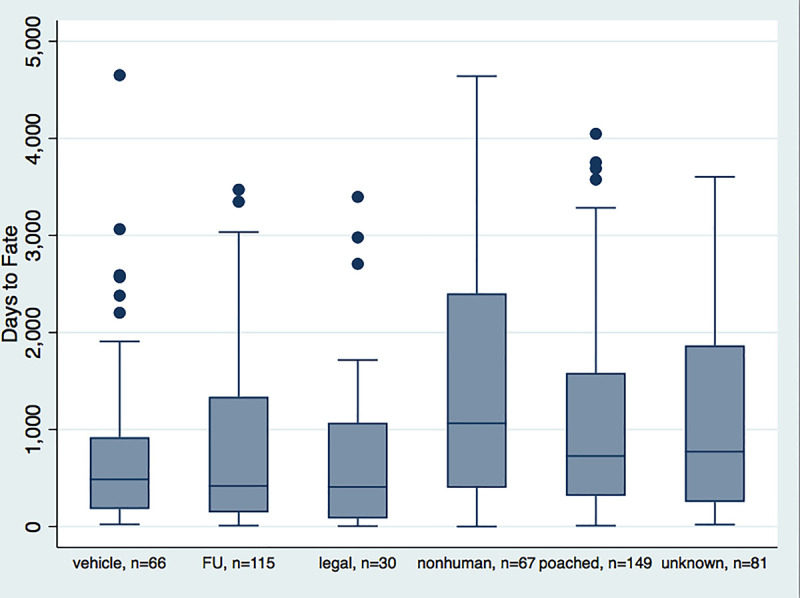
Days from collaring to death or disappearance (FU). (Fig 6 in S1 File).

Time after collaring until death or disappearance for 508 adult collared red wolves was 1009 ± 45 days SE (deaths only mean = 1067 ± 52 SE, range 0–4,651 days) (Fig 6). Nonhuman COD had the longest time in the wild and legal the briefest (Fig 6). There were significant differences between FU and all CODs (ANOVA F(1,506) = 5.93, p = 0.015) and between anthropogenic CODs (legal, vehicle, poached) and nonhuman COD (ANOVA F(1,310) = 13.30, p < 0.001). Out of 115 wolves classified as FU, 92 disappeared 100–3,471 days after collaring, 16 wolves between 41–100 days, 6 wolves between 20–40 days, and only one at 9 days after collaring. Time to disappearance and time to death from legal and vehicle were very similar (Fig 6).

## Discussion

Traditional survival analyses do not accurately represent marked animals that disappear (FU) since most are censored and not included in outcomes. Our results for risk of various causes of death (COD), when compared to previous published estimates, support the need for including unknown fates in risk estimates. Legal risk would have been overestimated using only a known fate model, leading to other imperfectly reported CODs being underestimated, first predicted by [28]. By summing known and unknown fates, our risk from anthropogenic causes is higher at 0.78 (scenario 1), 0.83 (scenario 2) and 0.85 (scenario 3) than previous estimates of 0.61 [17] and 0.75 [10]. The last corrected estimate (conducted with our same method) using data through 2007 [28] was slightly lower than ours at 0.77, suggesting either that uncertainty in both estimates make the two entirely overlapping despite our inclusion of 11 additional years of data or possibly that there has been a slight increase in anthropogenic mortality since that time. Ultimately, anthropogenic mortality reduces the probability of a self-sustaining population, the goal of the USFWS red wolf recovery plan, and could ultimately cause the extinction of the species in the wild in the absence of intervention [48]. Human CODs also violate the Endangered Species Act prohibitions on take.

Many studies show wolves face high levels of anthropogenic mortality [19, 28, 34, 49], however the persistence of some carnivore populations in areas of high human density show human-carnivore coexistence with minimal killing is possible under certain conditions [50, 51]. For example, Carter et al. found that tigers and humans co-existed even at small spatial scales, likely because tigers adjusted their activity to avoid encounters with people [51]. The same was shown for gray wolves in Minnesota [52]. In North America, Linnel et al., found that carnivore populations increased after favorable policy was introduced (e.g., ESA protections), despite increases in human population density [53]. Finally Dickman et al., (2011) assessed how people changed their behavior, adopting non-lethal methods for coexistence through financial incentives [54].

We estimated total poaching at 0.51–0.635 of 508 collard, adult red wolves, with cryptic poaching alone accounting for 0.028–0.293 (5–46% of total poaching). Our most conservative estimate for the risk of total poaching at 0.51 is also higher than any previously published risk estimate for red wolves, which ranged from 0.26 [47] to 0.44 [28]. Moreover, our less conservative scenarios estimate cryptic poaching at 0.54 and 0.63, similar to the Wisconsin estimate of 0.50 [28] and the Scandinavian estimate of 0.66 [33]. All three of our red wolf cryptic poaching scenarios lead to very similar total poaching risk, suggesting that regardless of the scenario, wolves in NENC are at higher risk from poaching than any other COD. This supports previous work that suggested poaching might be the cause of decline of this introduced population compared to success in the reintroduced gray wolf population in Yellowstone [10, 55]. To our knowledge poaching of red wolves is higher than any other wolf population measured thus far.

We detected an increase in risk of poaching and disappearances during the late fall hunting seasons, similar to other studies that have shown population declines during this same season in red wolves [10], gray wolves [34], and coyotes [56]. Just before fall hunting season, agricultural fields in this region, which make up approximately 30% of the red wolf recovery area [24], are typically cleared of protective cover for wolves [10]. This pattern is quite different from patterns during regulated summer hunting for turkeys from April–May when fields remain covered, vegetation is in leaf, and poaching is significantly lower than during the fall/winter hunting season. Although any type of hunting can mean more opportunity for poaching, the lack of an increase in red wolf poaching during the turkey season suggests the hypothesis that other types of hunters are implicated in red wolf poaching. The number of hunting licenses has increased every year in each of the five counties [57] and additional hunters on the landscape could mean more opportunities for poaching. In our study, all other mortality risk for collared, adult red wolves decreased or remained the same during legal fall/winter hunting seasons on other wildlife.

Adult, collared, red wolves also disappeared (FU) more during hunting seasons, suggesting a possible relationship with poaching. This finding is also consistent with the assumption that many FUs result from cryptic poaching (scenario 2). We estimated FU as cryptic poaching, but many readers will wonder if FU might not be largely emigrants out of NENC or transmitter failures followed by deaths of other causes in which carcasses were never recovered, rather than cryptic poaching. We draw on research with Wisconsin gray wolves and Mexican wolves [34, 58, 59] that provided numerous independent lines of evidence that the majority of FU could not be emigrants nor transmitter failures. First and most importantly, the gray and Mexican wolf studies demonstrated that rates of FU changed with policies on legal killing, which could not plausibly have caused transmitter failures. Therefore, transmitter failure is not likely to explain red wolf FU when it did not for gray and Mexican wolves. The USFWS uses Telonics VHF radio collars with an expected battery life of 6 or more years. Might a difference in ecosystem or latitude play a role in transmitter failure in NENC? Lower temperatures are associated with battery failures, yet the seasonal pattern of FU in NENC matches that of poaching, not the annual low temperatures that occur in the months of January through March in NC. Also, average low temperature in NC for this period is between -1.1–4.4°C, while WI averages between -17.8 –-8.9°C making transmitter failure due to low temperature even less plausible. Also, battery life would seem to play a greater role if FU occurred long after collaring. Contrary to this expectation, FU (808 ± 84 SE days) was much shorter than nonhuman COD (1450 ± 154 SE days). Second, if FU were largely made up of emigrants, these individuals would likely have died on roads in the densely settled counties beyond the NENC and some of these would have been reported to law enforcement. Even if emigrant collared red wolves escaped the NENC peninsula, some would have been found by citizens who presumably would have reported their observations to authorities. The intensity of monitoring this small population makes such emigration by collared animals hard to miss. No such cases are known to us and USFWS data show FU locations clustered amongst other CODs rather than along the edges of the RWRA. Also, dispersal of radio-collared red wolves seems unlikely in an ecosystem with low saturation and abundant, frequent vacancies in territories. Finally, our scenarios encompass non-poaching related explanations for FU and yet total poaching exceeds all other CODs given the known fates and all outcomes we considered.

Some of this poaching, whether cryptic or not, is sometimes attributed to mistaken identity. Since red wolves were reintroduced, coyotes have migrated into eastern NC and have been subject to intense shooting and trapping control efforts by regulated hunting [13]. Efforts therefore have been made to limit those occurrences of red wolf killing through regulation changes. Current NC state hunting regulations in the five-county red wolf recovery area restrict coyote hunting to daytime hours only, year-round, and with a permit [60]. However, in 2018 alone, 1752 coyote hunting permits were issued to both in-state and out-of-state residents, of which only 23% of recipients lived in the 5 county RWRA, suggesting a large number of hunters from outside the area are targeting coyotes while also hunting other species. Further studies or USFWS investigation into the connection of coyote hunting alongside other species during the winter hunting season would allow more insight into how this issue affects red wolves. This continued allowance of coyote hunting reflects a blind spot to the problem of poaching, and further efforts to limit this mistaken identity may be necessary. The ESA “Similarity of Appearance” clause allows the Secretary to treat any species as an endangered species if “the effect of the substantial difficulty (to differentiate between the listed and unlisted species) is an additional threat to an endangered or threatened species, and such treatment of an unlisted species will substantially facilitate the enforcement and further the policy of this Act.” (ESA, Sec.4.e). The 1987 amendments to the ESA do not define “knowingly” to include knowledge of an animals species or its protected status, rather it means the act was done voluntarily and intentionally and not because of a mistake or accident [44]. The McKittrick policy, which required a perpetrator must have known he/she was shooting a listed species before they could be prosecuted, was challenged successfully in federal district court [61], then overturned in the appellate court. Newcomer et al. consider Congressional intent in drafting the ESA was clear that harming a listed species would be a crime regardless of the intent or knowledge of the perpetrator, but the McKittrick policy weakens that protection [44].

Although there could be other factors affecting poaching numbers, political volatility [62] and evolving state policy appear to have affected killing of red wolves. For example, North Carolina House Bill 2006, effective January 1, 1995, allowed landowners to use lethal means to take red wolves on their property in Hyde and Washington counties in cases of defense of not only human life (as was always allowed by the ESA regulations) but also threat to livestock, provided the landowner had requested removal by the USFWS. After the announcement of this bill, four wolves were shot illegally over the course of Nov 1994 to Dec 1995 [62], the first known to be poached since reintroduction. It seems that both state and federal policy in NC have been less positive for red wolves and may partially explain why the population has declined to near-zero [63]. The USFWS acknowledged human caused mortality in their 2007 5-year review [26], was repeatedly warned about the problem of poaching while there was still time, with a population of 45–60 in 2016 [10, 43] and beginning in 2017 were notified of the problem of cryptic poaching being underestimated [28]. Those results were shared with the USFWS in public comments in 2018, an official peer review in 2019 and earlier warnings were repeated when comments on red wolves were solicited. It appears the ESA prohibition on take was not effectively enforced.

Our analysis relied on USFWS data for COD and disappearances because we could not verify these independently, which is a limitation of this study. Also, the USFWS has not used GPS collars on red wolves since 2013 and VHF location data includes death locations but not all wolf movements. Considering the recent decline in the population, we recommend using GPS collars whenever possible for more precise data collection with information that includes dates of collaring and last contact because of death or disappearance for all collared animals. Previous survival analyses on wildlife populations have not accounted for certain causes of death and disappearance, nor have they been able to model how individual wolves experience policy over time. While survival analysis can model changes in hazard rates, it does not verify why those changes take place such as possible social reasons for increased or decreased poaching. We recommend a time-to-event survival analysis that includes policy period as an intervention since increased poaching appears to be correlated with policy volatility [58]. There was a dramatic decline in the red wolf population in 2014–2015 and a more in-depth survival analysis may give more insight into this occurrence.

Because the large decline in population abundance takes the red wolf to the brink of extinction, the USFWS will need to implement policies that prohibit and prevent killing of red wolves, whether legal or illegal. Killing of red wolves for any reason, other than defense of human life or property, may actually lead to increases in poaching [36, 58]. Rather, programs that reward the presence of wolves can increase their value to local residents. One example of favorable policy related to carnivores was the wolverine program in Sweden, which offered rewards to Sami reindeer herding communities for having reproducing female wolverines on their communal lands [64, 65]. Across North America, success of carnivore populations has been linked to legal protections and enforcement [58, 66]. Therefore managing wolves successfully, by protecting wolves and encouraging acceptance, might be one way to generate support for their restoration [67]. In NENC policy has changed at both the state and federal level several times since reintroduction began and is currently being reviewed for further changes. This makes it difficult for residents to understand what current regulations are and can lead to confusion about what is allowed or not allowed with regard to killing red wolves. To maintain a positive working relationship with private landowners, USFWS might adopt and enforce policy that is consistent, clear, and protects wolves throughout the entire red wolf recovery area [68] even if it means standing up to illegal actors and communities that condone such law-breaking. Persuading the public to support red wolf recovery might be difficult if landowners resist ESA protections for the animals.

Because approximately 76% of the RWRA is private, and poaching occurs more on private land than public [41], any anti-poaching measure implemented must be proven to work on these lands. Knowledge of wolves’ locations through radio-collaring, trail cameras, and other methods simplify all aspects of management by allowing biologists to locate wolves for any reason including human conflict and should be a part of anti-poaching management. In the past, the USFWS had access to approximately 197,600 acres of private lands through both written and oral agreements. We believe that this has decreased in recent years, both because there are fewer wolves on private land, and because some landowners are no longer as supportive of the program or as willing to allow access, but we cannot quantify the change. A study on the social aspects of red wolf conservation with the local population is needed to understand attitudes toward red wolves and the motivations to poach them.

This study suggests aggressive interventions against poaching immediately would be needed if there is going to be any chance the remaining 7 red wolves [63] can create a self-sustaining population. In their most recent 5-year review completed in 2018, the USFWS recommended the red wolf retain its status as endangered under the ESA [69]. While there have been significant changes in the RWRA since reintroduction began, such as the migration of coyotes into the area and rising problems with poaching, the area still retains most of what made it appealing to reintroduction in the first place including low human density and suitable habitat of large areas of woody wetlands, agricultural lands, and protected areas. With the Red Wolf Adaptive Management Plan [70], the USFWS took an aggressive and successful approach to the encroachment of coyotes through sterilization [70] and should be as aggressive with poaching as mandated by the ESA. Implementing proven practices that prevent poaching or hasten successful reintroduction can reverse the trend of a decreasing NENC red wolf population and once again allow red wolves to thrive, not only in NENC but in additional future reintroduction sites.

## Supporting information

S1 File Data supporting tables and figures in this study. Data for Table 4 and Figs 4–6.
(XLSX)
